# What Shall Be Done with the Professional Midwife?1Read at the annual meeting of the Medical Society of the State of New York, January 28, 1902.

**Published:** 1902-03

**Authors:** Maurice J. Lewi

**Affiliations:** New York


### What Shall Be Done with the Professional Midwife?[1]

By MAURICE J. LEWI, M. D., New York.

FIGURES furnished by the Department of Health of the City of New York, show that during the year 1901, 80,735 births were reported, of which number 42,253 were reported by physicians, and 38,482 by midwives. The figures for the preceding years are very much the same as those just quoted.

Personal investigation through the medium of prominent physicians in the City of New York shows an undivided opinion based upon their experience that great evils are wrought to the community by reason of the incapacity and the negligence of these midwives.

It is a difficult matter to determine just how many cases of disease and deformity are properly chargeable to incompetency on the part of midwives at the bedside of the lying-in woman and at the birth of the child.

Suffice it, our hospitals and dispensaries are daily witnesses to the criminality which permits ignorant women to act as medical guardians at the birth of the child. The consensus of opinion, which holds this ignorance responsible for the many cases of ophthalmia neonatorum, and of ruptured perineums and of lacerated ora uterorum, is a conclusion well worthy of recognition, for it is unbiased and is gleaned from the actual experience of scientific men who lead in their several specialties.

By reason of the public lack of knowledge of the subject it has ever devolved on the medical profession to correct sanitary and health abuses, and as heretofore the laity expects the profession of medicine, through its accredited society or societies, to point out the remedy, when the law makers may be expected

1. Read at the annual meeting of the Medical Society of the State of New York, January 28, 1902.

to act. The difficulty up to this time in consummating a correction of this abuse, and a mitigation of this suffering has been due to a lack of unanimity on the part of the doctors as to method and manner.

Those opposed to interference state: "If you legalise these midwives, you permit uneducated persons to practise a branch of medicine, which practice requires more knowledge than the educated midwife can possess." The other faction has said: "Educate these midwives as they are educated in Europe, and there will be a mitigation of existing evils." The ideal method would, of course, be that which would make it legally necessary for one practising midwifery to be possessed of the knowledge essential to practise medicine generally. But such a view is utopian as long as the cities of this country are filled with a cosmopolitan population carrying with them to their new homes the old ideas regarding the need for midwives, and so long as no law other than a health board regulation, necessarily more honored in the breach than in the observance, is the only trip encountered by the would-be practitioner of midwifery, so long will there be women to be delivered by midwives in preference to doctors, and so long will there be midwives who are ignorant.

We can not perch ourselves on an eminence and look down upon the world and say: "You must come to our views, you who are suffering because of these abuses." But those of the profession who have all along hoped that this reckless practice of midwifery would be corrected by legislation calling for an abandonment of midwifery practice, by all not licensed to practise medicine, must cease from hoping for such an outcome. We are confronted with a condition not a theory. We know that the asylums for the blind are being crowded with children rendered sightless because of the neglect, criminal or otherwise, of these midwives. We know that dispensaries and hospitals are daily crowded with women who have been maimed and crippled because of the neglect or otherwise of this same class of people. We know that abortions are being daily committed by women advertising themselves as professional midwives. We know that the farming out of children is being carried on by many of this same class. Is it not high time that the medical profession of the State of New York should step in and demand an abatement of the conditions enumerated? Unitedly presenting our views to the legislature, is it not reasonable to expect that the legislature would come to the rescue? And would not a mitigation of these evils bring credit to the medical profession, if through its initiative this reform were accomplished?

And just as we would be honored for bringing this to pass, so are we today discredited, not by the laity, for they do not understand the situation, but by the thinking part of the profession which feels that each blind child, rendered so by failure on the part of the profession to intelligently regulate the practice of midwifery, is a crime for which the profession is responsible.

To quarrel about details thereby preventing the consummation of a corrective law, is as wrong as it would be for the various religious sects to quarrel with one another as to their creeds at the portals of heaven. The remedy:

Let the profession unite in petitioning the legislature to pass a law that on and after a given date no persons shall be permitted to practise midwifery (unless previously registered as having attended at least 10 cases of confinement), who is not licensed so to practise after examinations to be conducted on given topics, under rules and regulations to be formulated by the regents of the University of the State of New York, or by the State Board of Health, as you will.

The verbiage of this bill could be simple and still effective. It need not take up more space than a stick in a newspaper column. This caviling as to whose functions it is to attend cases of this character, the plaint of the sentimentalist in medicine that the profession is being insulted by recognising legally those who carry on this practise, and all other fancies, should be set aside and the great thought kept in mind that the public is entitled to a better condition of affairs, and that crime and physical wreck in the name of midwifery should cease.

149 W. 85TH STREET.

